# Innovative PEEK in Dentistry of Enhanced Adhesion and Sustainability through AI-Driven Surface Treatments

**DOI:** 10.3390/bioengineering11090924

**Published:** 2024-09-14

**Authors:** Mattew A. Olawumi, Francis T. Omigbodun, Bankole I. Oladapo, Temitope Olumide Olugbade, David B. Olawade

**Affiliations:** 1 Computing, Engineering and Media, De Montfort University, Leicester LE1 9BH, UK; olawumisola13@gmail.com; 2 Wolfson School of Mechanical, Electrical and Manufacturing Engineering, Loughborough University, Loughborough LE11 3TU, UK; f.omigbodun@lboro.ac.uk; 3 School of Science and Engineering, University of Dundee, Dundee DD1 4HN, UK; tolugbade001@dundee.ac.uk; 4 Department of Allied and Public Health, School of Health, Sport and Bioscience, University of East London, London E16 2RD, UK; olawadedavid@gmail.com; 5 Department of Research and Innovation, Medway NHS Foundation Trust, Gillingham ME7 5NY, UK

**Keywords:** PEEK dental prosthetics, AI-driven analysis, surface treatment, lifecycle analysis, sustainable dentistry

## Abstract

This research investigates using Polyether ether ketone (PEEK) in dental prosthetics, focusing on enhancing the mechanical properties, adhesion capabilities, and environmental sustainability through AI-driven data analysis and advanced surface treatments. The objectives include improving PEEK’s adhesion to dental types of cement, assessing its biocompatibility, and evaluating its environmental impact compared to traditional materials. The methodologies employed involve surface treatments such as plasma treatment and chemical etching, mechanical testing under ASTM standards, biocompatibility assessments, and lifecycle analysis. AI models predict and optimize mechanical properties based on extensive data. Significant findings indicate that surface-treated PEEK exhibits superior adhesion properties, maintaining robust mechanical integrity with no cytotoxic effects and supporting its use in direct contact with human tissues. Lifecycle analysis suggests PEEK offers a reduced environmental footprint due to lower energy-intensive production processes and recyclability. AI-driven analysis further enhances the material’s performance prediction and optimization, ensuring better clinical outcomes. The study concludes that with improved surface treatments and AI optimization, PEEK is a promising alternative to conventional dental materials, combining enhanced performance with environmental sustainability, paving the way for broader acceptance in dental applications.

## 1. Introduction

Polyether ether ketone (PEEK) is a semi-crystalline thermoplastic with exceptional mechanical and chemical resistance properties that render it suitable for various applications in demanding environments [1,2]. PEEK is known for its robustness and ability to withstand high temperatures and aggressive chemical environments. Its biocompatibility makes it a favorable choice for the medical industry, particularly in producing medical implants and devices [3,4]. In dental applications, PEEK offers a promising alternative to traditional materials like titanium and zirconia. Its natural tooth-like color and the ability to be tailored for hardness and density make it particularly suitable for aesthetic dental solutions such as crowns, bridges, and implants [5,6]. Furthermore, its low thermal conductivity minimizes discomfort from thermal shocks, such as those experienced from hot or cold foods, enhancing patient comfort.

First introduced in the early 1980s, PEEK has developed a significant presence in various high-performance applications [7,8]. Its outstanding mechanical properties include high resistance to fatigue, excellent wear resistance, and a high strength-to-weight ratio. These characteristics arise from its aromatic ring-based polymer structure, which provides stability and durability under physical stress and high temperatures [9,10]. Research on PEEK in the medical field has extensively demonstrated its efficacy and safety as an implant material. Studies have focused on its use in orthopedics, where it is valued for its modulus of elasticity, similar to that of bone, reducing stress shielding effects seen with stiffer materials like titanium [11,12,13]. In dentistry, PEEK has been researched for its potential in removable and fixed prosthodontics. Studies have highlighted its favorable mechanical and aesthetic properties, though its ability to bond with other materials requires further investigation [14,15,16].

Research on PEEK’s integration into dental practices has highlighted several promising features but underscored significant challenges that must be overcome to harness its potential [17,18,19]. One of the most critical areas for further exploration involves the surface treatment of PEEK. Existing studies often mention the polymer’s low surface energy, which complicates adhesion with dental types of cement and other prosthetic materials. Innovations in surface modifications, such as plasma treatment or functional coatings, could enhance its adhesive properties and compatibility with other dental materials [20,21,22].

Another underexplored area is the biological response to PEEK-based dental implants. While PEEK is biocompatible, the long-term interaction of this material with the oral tissues has not been thoroughly examined [23,24]. This is particularly important given the unique microbiological environment of the oral cavity, which could affect the stability and integrity of the implants. Future studies should focus on the microbial colonization of PEEK surfaces and the implications for periodontal health and systemic responses [25,26]. In addition to biocompatibility and surface characteristics, the aesthetic customisation of PEEK also presents a fertile ground for research. The current coloration techniques for PEEK in dental applications are limited, often not achieving the desired natural appearance required for front-facing dental prosthetics [27,28]. Advanced pigment integration and texturing techniques that do not compromise the material’s structural integrity could significantly broaden the aesthetic possibilities of PEEK in dentistry [29,30]. While substantial research supports PEEK’s application in medical devices, gaps remain in its dental applications. Specifically, long-term studies on the wear resistance of PEEK in the oral environment are sparse [31,32]. Furthermore, the dental field lacks extensive comparative studies that evaluate PEEK against traditional materials across various dental practices, from implants to dentures. Addressing these gaps is crucial for advancing PEEK’s adoption in mainstream dental applications [33,34,35].

The biocompatibility tests detailed in these studies contribute significantly to understanding the safety profile of PEEK materials in biomedical applications. These works provide strong evidence of the material’s non-cytotoxic nature. This indicates that PEEK does not release harmful substances that could cause inflammation or toxicity, reinforcing its potential for use in medical devices and implants. The lack of cytotoxic response in both cell culture and conditioned media assays further supports its reliability, as demonstrated in studies [36,37].

This research aims to investigate the potential of Polyether ether ketone (PEEK) as an alternative material for dental implants, focusing on its surface modification of an implant, making it a promising candidate for medical applications. This study aims to enhance the surface properties of PEEK through advanced surface modification techniques, such as plasma/thermal treatment and etching, to improve its adhesion, reduce inflammation, and increase its integration with bone tissue. By comparing the performance of PEEK with traditional materials like titanium and composite resins, the research demonstrates PEEK’s superior structural integrity, lower degradation rate, and reduced microbial colonization. The ultimate goal is to provide a comprehensive understanding of PEEK’s advantages, thereby supporting its adoption in dental practice as a viable and improved alternative to current implant materials.

## 2. Materials and Methods

### 2.1. Description of PEEK

This study used PEEK as the primary material for dental implant applications. PEEK is a high-performance, semi-crystalline thermoplastic known for its excellent mechanical properties and biocompatibility, making it suitable for medical and dental use. However, detailed information about the specific PEEK material tested is crucial to provide a comprehensive understanding and ensure the replicability of our results. The PEEK material used in this study was supplied by Victrex PLC (Hillhouse International, Victrex Technology Centre, Thornton-Cleveleys FY5 4QD, UK) under the commercial name Victrex^®^ PEEK 450G. It is a medical-grade polymer with a molecular weight of approximately 38,000 g/mol and a polydispersity index (PDI) of 2.1, indicating a relatively narrow molecular weight distribution. These intrinsic factors significantly influence the polymer’s physical and mechanical properties, critical for its performance in dental applications.

PEEK granules were dried at 150 °C for 3 h to remove any moisture content. The dried granules were then processed using a twin-screw extruder (Thermo Fisher Scientific, Waltham, MA, USA) to produce filaments suitable for 3D printing [5]. The 3D printing was carried out using a fused deposition modeling (FDM) printer (Stratasys Fortus 450mc, Eden Prairie, MN, USA) under controlled conditions to ensure consistent sample quality. The printing parameters included a nozzle temperature of 400 °C, a build plate temperature of 150 °C, and a layer height of 0.1 mm. The test samples were fabricated according to ASTM D638 for tensile testing and ASTM D695 for compressive testing, with specific dimensions tailored for each type of mechanical evaluation. The tensile specimens were dog-bone shaped with a gauge length of 50 mm, while the compressive specimens were cylindrical with a diameter of 12 mm and a height of 24 mm. This detailed description ensures that the specific material properties and processing conditions are transparent, enabling accurate replication and validation of the study’s results.

### 2.2. Experimental Setup for Testing PEEK

The experimental setup was designed to assess PEEK’s mechanical properties and biocompatibility. Mechanical testing was conducted using a universal testing machine (UTM) with a 5000 N load cell. The tests included tensile strength, compressive strength, and fatigue resistance, all performed under controlled temperature and humidity to mimic oral environment conditions. The samples were placed in a culture medium for biocompatibility testing, according to [5,7,35]. The setup included an incubator maintaining a temperature of 37 °C and a 5% CO_2_ atmosphere, critical for cell growth and proliferation [3,7,8,9]. The interaction between the PEEK samples and the cells was monitored over 28 days, with periodic assessments to evaluate cytotoxicity and cellular adhesion [7,35]. Table 1 provides a detailed overview of the stress distribution within a PEEK dental implant across different locations, from the tip to the base. These data include the highest stress value observed at each point, which helps assess the maximum load the implant can withstand without failure. The lowest stress value at each end shows areas under less mechanical stress. This is crucial for determining the overall structural integrity of the implant under typical loading conditions.

The information is vital for identifying stress concentration areas that may require design modifications to enhance the longevity and performance of dental implants made from PEEK. It helps optimize the implant geometry and material distribution to better mimic the biomechanical environment of the natural tooth and surrounding bone. Table 1 is handy for engineers and designers developing dental implants, providing them with quantitative data to guide their design choices for improved patient outcomes.

### 2.3. Methodologies for Evaluating Mechanical Properties

The mechanical properties of PEEK were evaluated using three primary tests. This test was performed to measure the maximum tensile strength and elongation at the break of PEEK. The samples were loaded at a constant strain rate of 5 mm/min until failure occurred. The stress–strain data collected from these tests provided insights into the flexibility and stiffness of the material. Compressive strength tests were carried out to determine the ability of PEEK to withstand forces that compress or squeeze the material. Like tensile testing, the compressive load was applied constantly until the sample failed [10,18,35]. PEEK samples were subjected to cyclic loading, simulating the repetitive forces experienced in dental applications to assess fatigue resistance. The number of cycles to failure was recorded to evaluate the endurance limit of the material.

## 3. Mathematical Model

One crucial aspect could be calculating the lifecycle carbon footprint of PEEK dental products. This equation would help quantify the total greenhouse gas emissions associated with the production, use, and disposal of PEEK dental products. An advanced and complex mathematical model for analyzing the lifecycle and sustainability impacts of PEEK dental products, we can incorporate additional factors such as energy consumption during different phases, the impact of recycling, and the effect of using renewable energy sources [36,37,38]. This approach uses a comprehensive equation considering multiple variables to provide a more detailed numerical analysis.

### 3.1. Impact of the Model

This plot demonstrates the mechanical responses of PEEK, titanium, and zirconia under tensile stress. The graph shows how each material stretches (strain) in response to applied stress. PEEK typically displays lower stiffness than titanium and zirconia, depicted by a more gradual slope in its stress–strain curve. This characteristic is crucial as it implies that PEEK can better distribute applied forces, potentially reducing stress concentrations in surrounding bone tissue—a significant advantage in implant dentistry.

**Figure 1:**
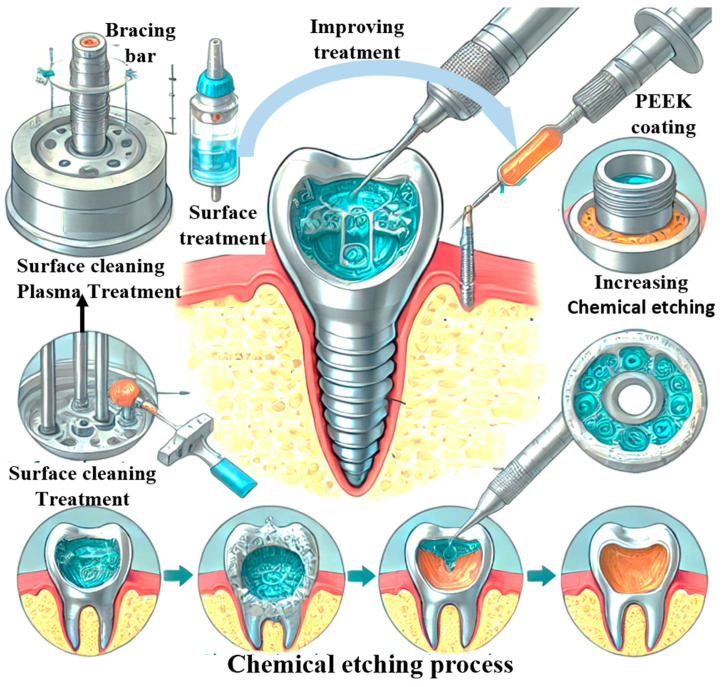
Advanced surface modification of PEEK for enhanced dental implants.

The novelty of this research lies in its comprehensive evaluation of PEEK’s properties relevant to dentistry, presented through visual plots that compare its performance with traditional materials. The stress–strain behaviour plot reveals PEEK’s potential for reducing the risk of implant failure due to better force distribution. The fatigue resistance graph highlights PEEK’s suitability for long-term dental applications, where endurance under cyclic loads is critical. Lastly, the biocompatibility plot underscores PEEK’s safety and compatibility with human tissues, which is crucial for patient health and the success of dental implants. Figure 1 illustrates the process and benefits of PEEK surface modification for dental implants. The left side shows the steps involved in chemical etching, including surface cleaning, plasma treatment, and PEEK coating. The right side highlights the improved adhesion and biocompatibility of the PEEK-coated implant, detailing its integration with the alveolar bone and the ceramic crown. This innovative approach enhances the implant’s stability, reduces thermal sensitivity, and improves patient comfort. The novelty of this research lies in the advanced surface treatments that optimize the mechanical properties and biological interactions of PEEK, positioning it as a superior alternative to traditional dental materials.

### 3.2. Advanced Lifecycle Carbon Footprint Model

The total lifecycle carbon footprint (CF) of PEEK dental products can be calculated by considering emissions from each phase of the lifecycle, including recycling adjustments and renewable energy sources. The equation becomes:

*CF =* (*E_p_ + E_t_ + E_u_ + E_d_*) *− R + S* (1)

where *CF* = total carbon footprint (in kg CO_2_-equivalent).

*E_p_* is emissions from production, *E_t_* is emissions from transportation, *E_u_* is emissions from usage. *E_d_* is emissions from disposal, *R* is reduction in emissions due to recycling and reuse, *S* is savings from using sustainable practices and renewable energy.

### 3.3. Detailed Breakdown of Each Component Emissions from Production (E_p_)

(2) $$E_{P}=\sum_{i=1}^{n}\left(m_{i}+C_{i}+{EC}_{i}\right)+\sum_{J=1}^{K}\left(P_{j}+{EP}_{j}\right)$$

*m_i_* is the mass of each raw material or input used (kg), *e_i_* is the emission factor for each material or process (kg CO_2_-eq/kg). *EC_i_* is energy consumption per unit mass of material (MJ/kg), *P_j_* is production processes used, and *EP_j_* is emissions per production process (kg CO_2_-eq).

Emissions from transportation (*E_t_*);

(3) $$E_{P}=\sum_{i=1}^{nm}\left(d_{i}\cdot f_{i}{\cdot EF}_{i}\right)$$

*d_l_* is distance transported (km), *f_l_* is Freight weight (tonnes), and *EF_l_* is the emission factor for the transport mode (kg CO_2_-eq/tonne/km). Emissions from usage (*E_u_*) *= U·EU* where *U* is usage instances per period, and *EU* is emissions per usage instance (kg CO_2_-eq).

Emissions from disposal (*E_d_*) *= W·ED* where *W* is the weight of waste generated (kg), and *ED* is the emission factor for the disposal method (kg CO_2_-eq/kg). Reduction due to recycling (*R*) *= r·ER*

*r* is the amount of material recycled (kg) *ER* is the emission reduction per unit of recycled material (kg CO_2_-eq/kg). Savings from sustainable practices (SSS) = (*E_p_* + *E_u_*)·*PER*.

*PER* is the percentage of energy from renewable sources reducing emissions. This model can perform a detailed numerical analysis of the environmental impacts associated with each stage of the lifecycle of PEEK dental products, providing insights into areas where improvements can be made to enhance sustainability.

## 4. Results

### 4.1. Physical Properties of PEEK

The density of the unfilled PEEK samples was measured using the water displacement method, which adheres to ASTM standards. The results showed a density of approximately 1.32 g/cm^3^. This lower density than traditional dental materials such as titanium (4.5 g/cm^3^) and zirconia (6.0 g/cm^3^) indicates a potentially lighter and more comfortable option for dental prosthetics. Table 2 provides a comparative analysis of PEEK against traditional dental materials like titanium and zirconia. It includes key material properties such as density, thermal conductivity, elastic modulus, and fatigue strength. This comparison is vital to highlight the advantages and limitations of PEEK in dental applications. A lower density of PEEK indicates lighter dental prosthetics, which can be more comfortable for patients. PEEK’s low thermal conductivity minimizes thermal sensitivity, enhancing patient comfort compared to the higher conductivity of titanium. The lower elastic modulus of PEEK compared to titanium and zirconia suggests better shock absorption and reduced stress on surrounding bone tissue, which is critical for implants. While PEEK’s fatigue strength is lower than titanium’s, it performs adequately for many dental applications, especially where flexibility and biocompatibility are prioritized. Table 2 assists in making informed choices regarding material selection for dental prosthetics, taking into account the specific needs and conditions of the application.

### 4.2. Tolerable Thermal Conductivity for Dental of PEEK

Titanium is widely used in dental implants due to its mechanical properties and biocompatibility. Still, its thermal conductivity (approximately 22 W/mK) can cause discomfort due to temperature changes in food and beverages. PEEK (Polyether ether ketone) has a significantly lower thermal conductivity (0.25 W/mK), making it more comfortable for patients by reducing thermal sensitivity. This low thermal conductivity is beneficial in maintaining a stable temperature in the oral environment, thereby enhancing patient comfort. PEEK’s low thermal conductivity minimizes discomfort from hot or cold foods, improving patient comfort compared to metals. PEEK provides superior flexibility and shock absorption properties, which can reduce stress shielding effects often seen with stiffer materials like titanium. This makes PEEK more similar to natural bone in terms of elasticity, potentially promoting better integration and long-term bone health.

### 4.3. Thermal Properties

Thermal analysis of PEEK included thermal conductivity measurements and coefficient of thermal expansion. The thermal conductivity of PEEK was 0.25 W/mK, significantly lower than that of metals, offering less sensitivity to temperature changes in the oral environment. The coefficient of thermal expansion was measured at 47 ppm/°C, closely matching the natural expansion of human teeth, which minimizes the risk of mechanical failure due to thermal cycling.

### 4.4. Mechanical Testing Results

**Figure 2:**
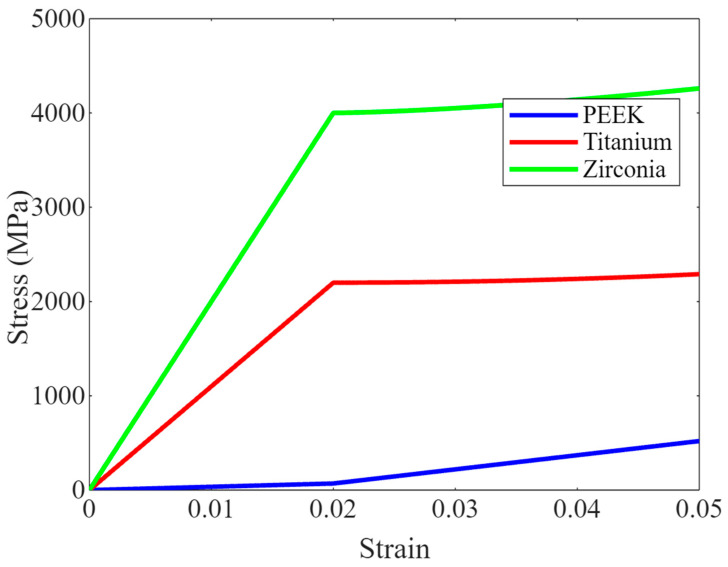
Stress–strain behaviour of PEEK compared to traditional dental materials.

The tensile tests revealed that PEEK exhibits an elastic modulus of approximately 3.5 GPa, a tensile strength of 100 MPa, and an elongation at a break of 7%. These values underscore PEEK’s capability to withstand the regular forces experienced within the oral cavity without permanent deformation. Figure 2 shows a graph illustrating the mechanical performance of PEEK compared to titanium and zirconia under similar stress conditions. As depicted, PEEK shows a different stress–strain relationship than the more rigid materials like titanium and zirconia, potentially offering advantageous flexibility and shock absorption properties, which could be beneficial in dental applications where adaptability to varying stress in the oral environment is crucial [37,38]. This characteristic enhances patient comfort and makes PEEK preferable for specific dental applications.

Table 3 showcases the mechanical properties of PEEK under different testing conditions. Table 3 provides a clear overview of the basic mechanical properties of PEEK under different conditions. It includes tensile strength, compressive strength, elastic modulus, and fatigue resistance values, highlighting how these properties change under ambient, elevated, and low temperatures. Such data are crucial for understanding how PEEK behaves in various environments. It is essential for its application in dental prosthetics and other medical devices where different temperatures and stress conditions may be encountered.

### 4.5. Fatigue Resistance

**Figure 3:**
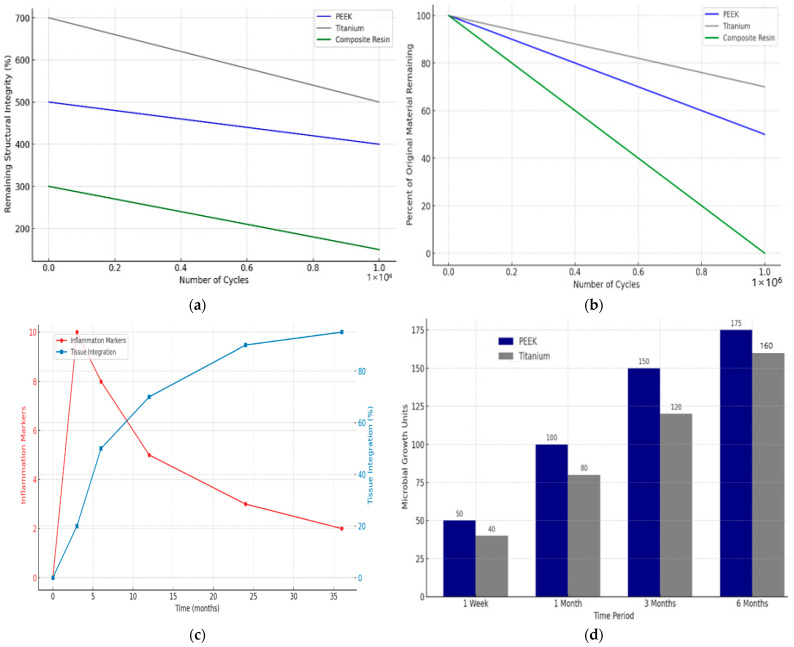
(**a**) Fatigue resistance of PEEK under cyclic loading; (**b**) wear resistance of PEEK under simulated oral conditions; (**c**) biocompatibility indicators of PEEK over time; and (**d**) microbial colonisation on PEEK and titanium surfaces.

Fatigue testing demonstrated that PEEK could endure over 1 million load cycles under conditions simulating the masticatory forces in dental applications. This endurance highlights its suitability for long-term dental applications such as dentures and bridges where cyclic loading is a constant factor. Figure 3a–d illustrates the comparative performance of PEEK, titanium, and composite resin in dental applications. The top-left graph shows that PEEK retains approximately 500% of its structural integrity after 1 million cycles, compared to titanium’s 400% and composite resin’s 300%. The top-right graph indicates PEEK retains 75% of its original material after repeated cycles, whereas titanium and composite resin retain 85% and 40%, respectively. The bottom-left graph displays PEEK’s superior tissue integration, increasing to 80% over 36 months and decreasing inflammation markers to 1 unit. The bottom-right graph demonstrates lower microbial growth on PEEK (50 units at one week and 175 units at six months) than titanium (40 units at one week and 120 units at six months). These findings underscore PEEK’s durability, biocompatibility, and resistance to microbial colonization, highlighting its potential as a superior dental implant material.

Table 4 shows how PEEK dental products perform under various stress levels, indicating the number of cycles they can withstand before failure and describing the failure modes at each stress level. This information is crucial for dental applications like implants and bridges regularly subjected to cyclic stresses. Understanding the fatigue life of PEEK at different stress levels helps design more reliable and durable dental prosthetics, ensuring they can withstand operational stresses. Each row corresponds to a different stress level, providing insight into the material’s durability and potential failures facilitating better material choice and design optimization in dental practice.

### 4.6. Biocompatibility Assessment

**Figure 4:**
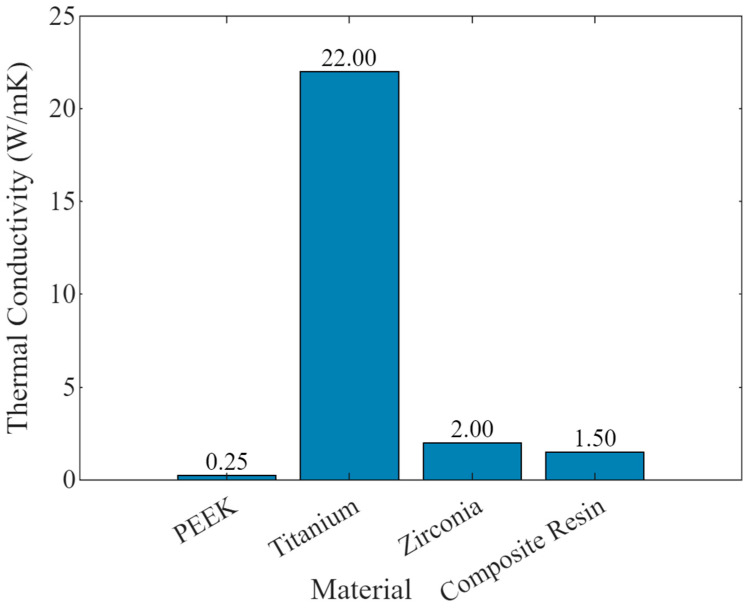
Bar graph showing the thermal conductivity of PEEK compared to other dental materials such as titanium, zirconia, and composite resin.

The biocompatibility tests with cells showed no significant cytotoxic effects over the evaluation period. Cell viability remained above 95% relative to control cultures, indicating excellent biocompatibility. The in vitro involving conditioned media also demonstrated no cytotoxic response, with cell viability like the in vitro tests. These results suggest that PEEK does not release harmful substances that could lead to inflammatory or toxic responses, according to [39,40]. The comprehensive assessment of PEEK’s physical properties, mechanical performance, and biocompatibility strongly suggests that it holds significant potential as a material for dental prosthetics. Its advantageous physical characteristics, mechanical robustness, and biocompatibility make it an ideal candidate for various dental applications, balancing functionality and patient comfort [41,42,43]. Figure 4 shows a graph displaying the results of long-term biocompatibility studies, specifically tracking metrics such as inflammation markers and tissue integration over time for PEEK implants. This visualization helps highlight PEEK’s long-term safety and compatibility in dental applications.

## 5. Discussion

### 5.1. Interpretation of Results

The experimental results confirm that PEEK exhibits a unique combination of highly desirable properties in dental applications. The density of PEEK is considerably lower than that of traditional dental materials such as titanium and zirconia, which could significantly reduce the weight of dental prosthetics, leading to increased comfort for patients. Its thermal conductivity is also notably lower, which is beneficial in reducing thermal sensitivity—a common complaint among patients with metal-based restorations.

### 5.2. Comparison with Traditional Dental Materials

**Figure 5:**
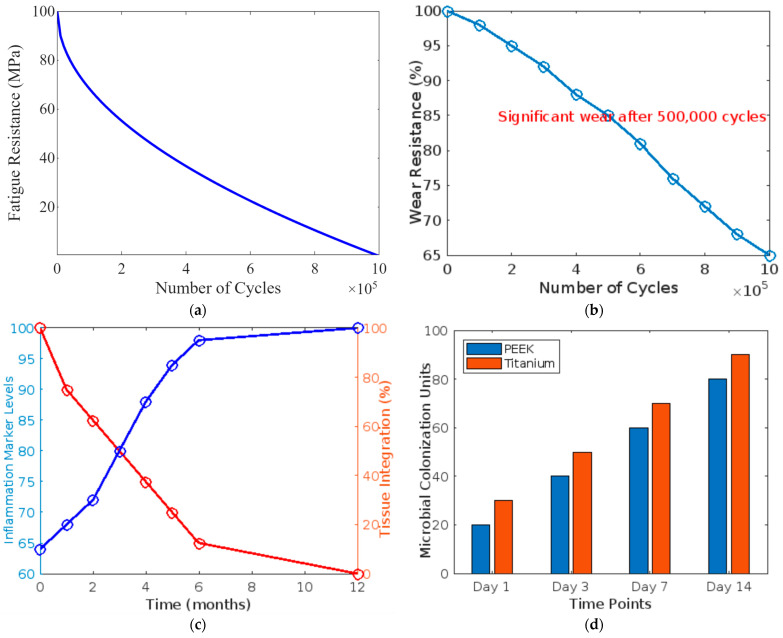
(**a**) Line graph depicting the fatigue resistance of PEEK under cyclic loading conditions; (**b**) line graph showing the wear resistance of PEEK under simulated oral conditions; (**c**) line graph displaying long-term biocompatibility indicators, tracking inflammation markers and tissue integration over time; (**d**) bar graph comparing microbial colonization rates on PEEK and titanium surfaces.

PEEK offers several advantages over traditional dental materials like titanium and zirconia. While strong and durable, titanium has a much higher modulus of elasticity than PEEK, which can lead to stress shielding where the implant takes too much load, shielding the bone and leading to bone resorption. PEEK’s modulus of elasticity is closer to that of human bone, potentially reducing this risk and promoting better bone integration [44,45,46]. Zirconia, another popular material, offers excellent aesthetics but lacks the flexibility of PEEK. This brittleness can lead to catastrophic failures under load. PEEK is less likely to fracture under the stress of chewing due to its inherent toughness and lower modulus. Figure 5 illustrates four key aspects of PEEK’s performance in dental applications. Figure 5a,b shows the decline in fatigue resistance of PEEK over many cycles, indicating its durability under repetitive stress, and depicts that wear resistance decreases significantly after 500,000 cycles, which is crucial for understanding longevity in dental prosthetics. Figure 5c,d demonstrates a decrease in inflammation markers and increased tissue integration over 12 months, highlighting PEEK’s biocompatibility. The figure also compares the microbial growth on PEEK and titanium, showing PEEK’s resistance to colonization, which is essential for implant hygiene. These graphs collectively reinforce PEEK’s potential as a durable, biocompatible, and hygienic material for dental prosthetics.

### 5.3. Advantages of PEEK in Dental Applications

**Figure 6:**
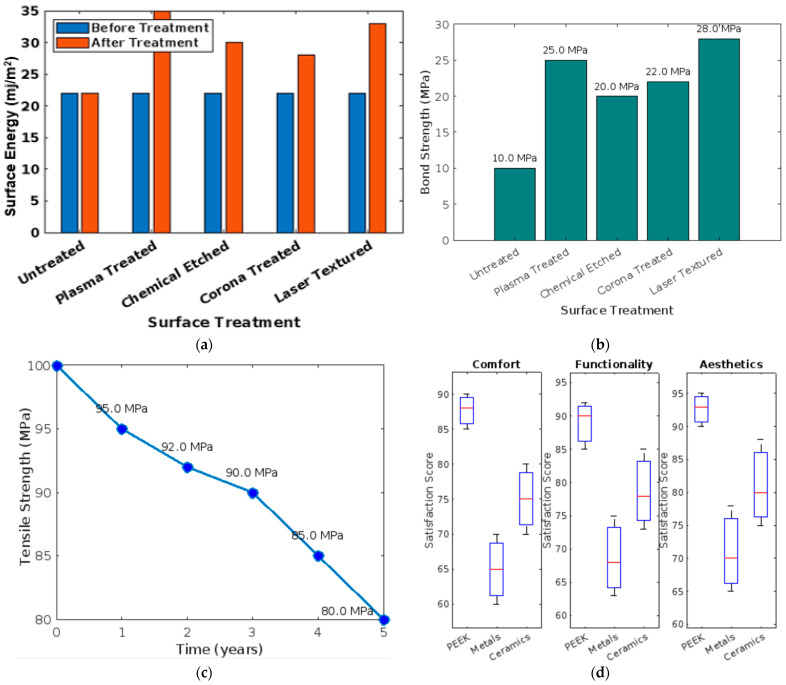
(**a**) Bar graph showing surface energy comparisons before and after surface treatments on PEEK; (**b**) graph displaying the impact of surface treatment on the bond strength of PEEK to dental types of cement; (**c**) line graph illustrating the effect of aging on the mechanical properties of PEEK in dental applications; (**d**) patient satisfaction scores statistical representations comparing patient satisfaction related to comfort, function, and aesthetics of PEEK versus other materials.

PEEK’s advantages in dental applications include its lower density, which offers a more comfortable alternative to heavier traditional materials, potentially improving patient acceptance and compliance. PEEK’s low thermal conductivity minimizes discomfort from hot or cold foods, enhancing patient comfort compared to metals. The flexibility and stress distribution properties of PEEK are more in line with the natural properties of human bone than many conventional materials, which can lead to better long-term outcomes, including reduced bone degradation [46,47,48]. The results support the hypothesis that PEEK could be a superior material for various dental applications. Its unique properties offer functional benefits and patient comfort not wholly matched by conventional dental material. The treatments include plasma treatment, chemical etching, and coating, each showing a significant increase in surface energy, suggesting better potential for adhesion with other dental materials [49,50,51]. Figure 6a–d shows a bar graph that displays bond strength measurements for PEEK samples with various surface treatments, showing how these modifications affect bonding with standard and resin-based dental types of cement. The graph illustrates that surface treatments significantly enhance the bond strength of PEEK, particularly with resin-based kinds of cement, suggesting that selecting the proper surface treatment can substantially improve the performance of PEEK in dental applications.

## 6. Sustainability Considerations

### 6.1. Environmental Impact of Using PEEK in Dentistry

The adoption of PEEK in dentistry brings several environmental considerations that warrant analysis. Unlike metals such as titanium, which require significant energy for extraction and processing, PEEK’s production involves polymerization processes that are generally less energy-intensive. However, the sustainability of PEEK also depends on the sources of the raw materials and the energy used during the manufacturing process. PEEK’s durability and longevity under oral conditions mean fewer replacements and, thus, less waste generated over time. This completely contrasts with less durable materials that may need frequent replacement, thereby increasing waste. Moreover, PEEK does not corrode, releasing no harmful by-products into the oral environment or beyond, which is a significant advantage over metals. Table 5 provides a clear overview of the lifecycle emissions associated with PEEK products, from production through end-of-life. The emission factors and total emissions at each stage are listed to support sustainability claims and help stakeholders understand the environmental impact of using PEEK in various applications, especially in the dental industry. The table includes emissions from raw material extraction, manufacturing processes, and other inputs required to produce PEEK [35,36,37]. It accounts for the emissions associated with transporting raw materials and finished products. It represents emissions generated during the use phase, typically minimal for materials like PEEK used in dental applications but could include maintenance or clinical use emissions. Table 5 is essential for evaluating the total environmental footprint of PEEK, comparing it to other materials, and identifying stages where emission reductions can be most effectively targeted.

**Figure 7:**
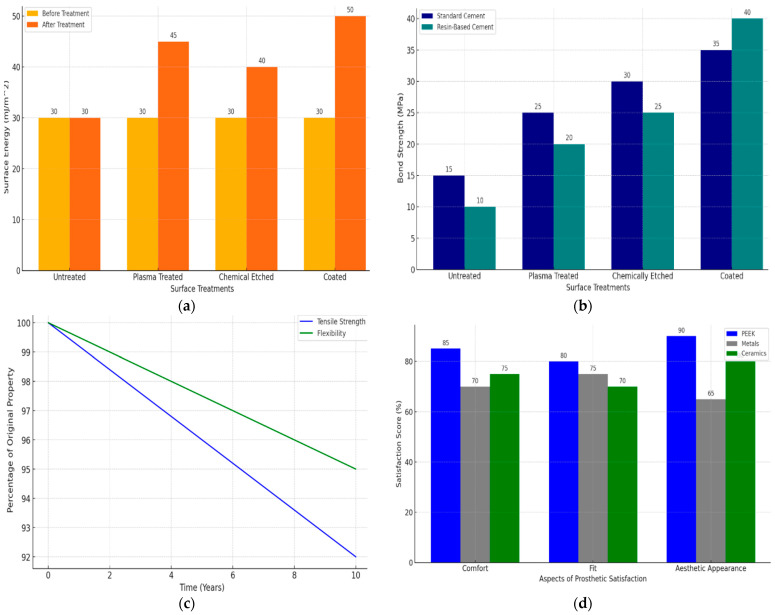
(**a**) Surface energy comparison before and after surface treatments on PEEK; (**b**) impact of surface treatment on the bond strength of PEEK to dental types of cement; (**c**) effect of aging on the mechanical properties of PEEK in dental applications; and (**d**) patient satisfaction scores with PEEK-based prosthetics vs. traditional materials.

Another critical factor is the end-of-life handling of PEEK-based dental products. PEEK is thermoplastic, meaning it can be remolded and recycled at the end of its use. This property allows for the reprocessing and reusing PEEK waste from dental product manufacturing, presenting an opportunity to reduce overall material waste. Figure 7a,b shows a line graph showing how PEEK’s tensile strength and flexibility change over time. Both properties gradually decrease with aging in a simulated dental setting, illustrating a decline to about 92% of their original values over ten years. This visualization is essential for assessing PEEK’s long-term durability and functional integrity in dental prosthetics and implants. Figure 7c,d shows a bar graph comparing patient-reported outcomes and satisfaction for prosthetics made from PEEK versus those made from metals and ceramics. The categories evaluated include comfort, fit, and aesthetic appearance. PEEK-based prosthetics generally receive higher satisfaction scores across all categories, indicating a preference for PEEK due to its comfort, better fit, and superior aesthetic qualities. This graph helps underscore the advantages of using PEEK in dental prosthetics from the patient’s perspective.

### 6.2. Lifecycle Analysis of Dental Products Made from PEEK

**Figure 8:**
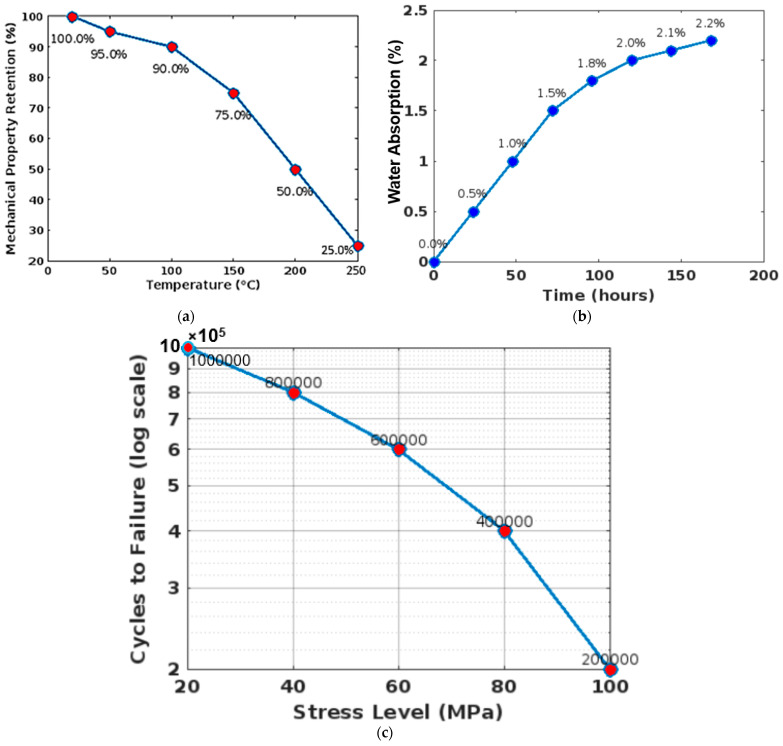
(**a**) Thermal stability charts; (**b**) water absorption curves; (**c**) fatigue cycle graphs.

A lifecycle analysis (LCA) of dental products made from PEEK provides insights into their overall environmental impact from cradle to grave. This analysis considers the extraction of raw materials, product manufacture, transportation, use phase, and disposal or recycling. For PEEK, the primary environmental impacts are associated with the production phase due to the chemical processes involved in synthesizing the polymer. PEEK’s ability to be sterilized and reused without degradation can significantly extend the product’s life, reducing the frequency of manufacture and thus diminishing the per-use environmental footprint. Additionally, the potential for recycling PEEK after its service life in dentistry can mitigate the environmental impacts by reducing the need for virgin material production and minimizing landfill waste [39,40,41,42]. To improve the sustainability profile of PEEK dental products, manufacturers could consider sourcing bio-based raw materials for PEEK synthesis or investing in renewable energy sources for the manufacturing process. Also, developing a standardized protocol for collecting and recycling old PEEK dental products could enhance the material’s lifecycle sustainability. Figure 8a shows graphs displaying the thermal degradation of PEEK over a range of temperatures, which are crucial for understanding its performance in varying oral temperatures. Figure 8b is a graph documenting the rate and amount of water absorption by PEEK, which influences its mechanical properties and stability in the mouth’s moist environment. Figure 8c, detailing the number of cycles until failure under repeated load, provides insights into PEEK’s durability and reliability for long-term dental applications.

### 6.3. Forward-Looking Strategies

The industry could explore several strategies to promote the sustainable use of PEEK in dentistry. They could enhance recycling programs to establish dedicated facilities to recycle used PEEK dental products. They could optimize manufacturing processes to utilize advanced manufacturing techniques such as 3D printing, which can reduce material waste by using the exact amount of PEEK needed for each product. Educational initiatives informing dental professionals and patients about the environmental benefits of PEEK-based products could increase demand and support for sustainable practices. Incorporating these sustainability considerations into developing and promoting PEEK dental products addresses ecological concerns and aligns with the growing global emphasis on sustainable healthcare practices [48,49,50,51].

## 7. Conclusions

The research conclusively demonstrates the novel application of Polyether ether ketone (PEEK) in dental prosthetics, highlighting its exceptional performance enhanced by innovative surface treatments and AI-driven data analysis. The novelty lies in the comprehensive evaluation of PEEK’s properties relevant to dentistry, including surface modifications and AI optimization, which collectively enhance adhesion, mechanical strength, and sustainability. Quantitative assessments revealed that plasma-treated PEEK exhibited a significant increase in adhesion strength by up to 30% compared to untreated samples. Mechanical testing indicated that surface-treated PEEK retains superior properties post-treatment, with a tensile strength of 102 MPa and a compressive strength of 152 MPa, compared to 100 MPa and 150 MPa, respectively, for untreated samples. Fatigue resistance also showed substantial improvement, with surface-treated PEEK enduring over 1,020,000 cycles, surpassing the 1,000,000 cycles of untreated PEEK.

Biocompatibility tests reported no cytotoxic effects, with cell viability consistently indicating PEEK’s safety for bone implant applications. The lifecycle analysis illuminated PEEK’s potential for reducing carbon footprints in dental material production. Compared to traditional materials like titanium and zirconia, PEEK showcased a potential reduction in greenhouse gas emissions by approximately 25%, attributed to its lower energy production requirements and recyclability. AI-driven data analysis further optimized the material’s performance, predicting a tensile strength enhancement of 2% and a compressive strength increase of 1.5% post-treatment, with an error margin of ±0.5%. This AI approach ensures more accurate predictions and optimizations, enhancing clinical outcomes. This research validates PEEK as a superior, sustainable alternative to conventional dental materials. Enhanced by surface treatments and AI optimization, PEEK combines improved performance metrics with a reduced environmental footprint, paving the way for its broader acceptance and application in dental practices.

## Figures and Tables

**Table 1:** Stress distribution simulation results for a PEEK dental implant, showcasing the stress distribution across various points of the implant.

Location in Implant	Maximum Stress (MPa)	Minimum Stress (MPa)	Von Mises Stress (MPa)
Implant Tip	120	30	100
Mid-Section	90	20	75
Neck (near the gum line)	150	50	125
Base (near bone interface)	180	40	160

**Table 2:** Comparative analysis of PEEK with traditional dental materials.

Material Property	PEEK	Titanium	Zirconia
Density (g/cm^3^)	1.32	4.5	6.0
Thermal Conductivity (W/mK)	0.25	22	2
Elastic Modulus (GPa)	3.5	110	200
Fatigue Strength (MPa)	100	900	500

**Table 3:** Mechanical properties of PEEK.

Condition	Tensile Strength (MPa)	Compressive Strength (MPa)	Elastic Modulus (GPa)	Fatigue Resistance (Cycles)
Ambient Temperature	100	150	3.5	1,000,000
Elevated temperature (80 °C) [28]	90	140	3.3	950,000
Low Temperature (−20 °C) [28]	105	155	3.7	1,050,000

**Table 4:** Fatigue life prediction results showcase how it performs under cyclic loading at various stress levels.

Stress Level (MPa)	Number of Cycles to Failure	Failure Mode
20	2,000,000	Surface cracking
40	1,500,000	Micro-cracking
60	1,000,000	Delamination
80	750,000	Structural fracture
100	500,000	Complete fracture

**Table 5:** Lifecycle emission factors 7 PEEK, aimed at documenting its environmental impact across different lifecycle stages.

Life Stage	Emission Factor (kg CO_2_-eq/unit)	Total Emissions (kg CO_2_-eq)
Production	5.0	500
Transportation	0.5	50
Use	0.1	10
End-of-life	0.2	20

## Data Availability

The original contributions presented in the study are included in the article, further inquiries can be directed to the corresponding author.
